# Learning to Recognize Unfamiliar Voices: An Online Study With 12- and 24-Month-Olds

**DOI:** 10.3389/fpsyg.2022.874411

**Published:** 2022-04-26

**Authors:** Adriel John Orena, Asia Sotera Mader, Janet F. Werker

**Affiliations:** ^1^Department of Psychology, University of British Columbia, Vancouver, BC, Canada; ^2^Department of Evaluation and Research Services, Fraser Health Authority, Surrey, BC, Canada

**Keywords:** voice recognition, speaker perception, infancy, indexical information, online study

## Abstract

Young infants are attuned to the indexical properties of speech: they can recognize highly familiar voices and distinguish them from unfamiliar voices. Less is known about how and when infants start to recognize unfamiliar voices, and to map them to faces. This skill is particularly challenging when portions of the speaker’s face are occluded, as is the case with masking. Here, we examined voice−face recognition abilities in infants 12 and 24 months of age. Using the online *Lookit* platform, children saw and heard four different speakers produce words with sonorous phonemes (high talker information), and words with phonemes that are less sonorous (low talker information). Infants aged 24 months, but not 12 months, were able to learn to link the voices to partially occluded faces of unfamiliar speakers, and only when the words were produced with high talker information. These results reveal that 24-month-old infants can encode and retrieve indexical properties of an unfamiliar speaker’s voice, and they can access this information even when visual access to the speaker’s mouth is blocked.

## Introduction

In face-to-face conversations, we can tell who is speaking by looking at whose mouth is moving. Given the intersensory redundancy between this visual information and the resulting vocal signals, it is tempting to dismiss voice recognition as a trivial skill. However, there are often situations in which a listener may not have access to visual information—for example, during auditory-only telecommunication, when facing away from the speaker, or when the speaker is wearing a face mask. Moreover, familiarity with a speaker’s voice has consequences for social communication and linguistic processing (see Creel and Bregman, 2011). Thus, examining how listeners recognize voices when visual facial information is partially occluded is important for our understanding of how humans process speech. In the current study, we approached this question by examining whether infants can detect a speaker change when the speaker’s face is partially occluded.

From an early age, humans are surprisingly adept at tracking highly familiar voices. Previous studies have reported that upon hearing their mother’s voice (and relative to hearing an unfamiliar woman’s voice), fetuses’ heart rate increased (Hepper et al., 1993; Kisilevsky et al., 2003), newborns preferentially sucked on a pacifier in a non-nutritive sucking procedure (DeCasper and Fifer, 1980), and 4-month-olds looked toward their mother (live: Spelke and Owsley, 1979; photograph: Orena and Werker, 2021). Infants can also differentiate between their father’s voice and an unfamiliar male voice (DeCasper and Fifer 1980; Ward and Cooper, 1999). These studies show that, in the absence of synchronous visual information, infants can match highly familiar voices to the identities of familiar individuals.

A related question is whether infants can discriminate and learn to recognize unfamiliar voices with the same ease. Indeed, processing voices from *unfamiliar* talkers is a separate and often more challenging task than processing *highly familiar* voices (Stevenage, 2017). Infants show more attentive and mature processing of speech spoken by highly familiar voices versus an unfamiliar speaker (Purhonen et al., 2004; Naoi et al., 2012). Unsurprisingly, with increased exposure to a certain set of speakers, adult listeners similarly improve at differentiating the voices of those speakers (e.g., Levi et al., 2011).

Research-to-date indicates that infants are highly adept at discriminating between unfamiliar voices, particularly when the pair of voices are highly distinct from each other (e.g., Floccia et al., 2000). Moreover, infants can pair male and female voices with male and female faces, respectively, by 8 months of age (Patterson and Werker 2003). Infants can also successfully discriminate between voices of same-gender pairs of speakers. In a series of studies, researchers reported that, after being habituated to an unfamiliar female voice, infants as young as 4 months of age dishabituated to a new unfamiliar female voice (Johnson et al., 2011; Fecher and Johnson; 2018, 2019).

Of note, much of the infant work on processing and learning unfamiliar voices has been limited to tests of discrimination, rather than recognition. A recent study by Fecher et al. (2019) revealed that even 16.5-month-old infants have difficulty in learning the voices of unfamiliar speakers. In their task, infants were shown pairings of two voices and their identities (either cartoon characters or talking human faces). When the pair of voices involved one male and one female speaker, infants showed learning of the two voices in a preferential looking procedure experiment. However, when the pair of voices involved two female speakers, infants showed no recognition of either voice, suggesting that learning to recognize unfamiliar face-voice pairings may be a challenging task.

Like adults, certain factors appear to modulate infants’ talker processing abilities. For instance, it is now well-established that listeners are better at learning others’ voices when they are speaking a familiar versus an unfamiliar language (Goggin et al., 1991). Some studies indicate that this effect is a function of phonological processing (Perrachione et al., 2011; Kadam et al., 2016). Yet, even long-term systematic exposure to a language appears to facilitate talker processing (Orena et al., 2015), suggesting that benefits to talker processing could emerge prior to comprehension of the language. Indeed, even infants show a language-familiarity benefit to voice discrimination (Johnson et al., 2011). Recently, Fecher et al. (2019) found that 4-month-olds could discriminate between female voices speaking a familiar language, but not when speaking an unfamiliar language. These findings show that phonetic and indexical information is integrated early in speech processing.

In the current study, we examined the nature of early talker processing skills by tackling two research questions. First, we examined the voice recognition abilities of young children. We followed up on work by Fecher et al. (2019) to investigate whether young children will show voice recognition of unfamiliar voices when cognitive demands are eased. In Fecher et al. (2019), infants learned the face−voice pairings during a six-trial training phase before being tested on their recognition of the pairings during a two-trial test phase. In our study, infants were taught the face−voice pairings and then tested on their recognition of the face−voice pairings within the same trial. Given that 16.5-month-olds in Fecher et al. (2019) still had difficulty in learning the voices of unfamiliar speakers, we chose a higher age range (i.e., 24-months-old) to examine if it is more stable at this age point. We also tested a young age range (i.e., 12-month-olds) to examine whether young infants would succeed in this modified task. Importantly, in our work, in the test phase the faces were partially occluded.

As a secondary question, we asked whether, like for adults, phonetic content influences the voice discrimination abilities of young children. Work by Andics et al. (2007) found that segmental information contributes to ease of voice discrimination for adults. In their study, listeners heard blocks of various consonant-vowel-consonant words and had to decide whether the word they heard was produced by the same voice as the preceding word or by a different voice. Results indicated that certain segments—particularly segments that were more sonorant (e.g., [m], [s])—helped listeners discriminate voices more than other segments. Here, we examined whether certain phonetic segments in speech are helpful for infants to discriminate between voices.

## Experiment 1

### Methods

#### Participants

We recruited families through two avenues. First, as part of the Early Development Research Group at the University of British Columbia, we contacted families in our database to take part in the online study. To supplement this recruitment effort, we also publicly posted our study on the *LookIt* website. Data collection occurred between August 2020 and July 2021.

We recruited infants from two age groups. Thirty-one 12-month-old infants participated in the study, but five infants were excluded from the final sample because of technical issues (1), being too fussy during the session (2), and parental interference (2). Thus, we analyzed data from 26 12-month-old infants (mean age = 384 days; age range = 366–396; 12 girls and 14 boys). In addition, 38 24-month-old infants participated in the study. Eight infants were excluded from the final sample because of technical issues (2), being too fussy during the session (3), and for being above our age criteria (3). We analyzed data from the remaining 30 24-month-old infants (mean age = 784 days; age range = 732–774; 16 girls and 14 boys). Based on parent-report measures, all infants were exposed to English at least 90% of the time and had no known hearing or language impairments.

#### Study Platform

The study was conducted in participants’ homes, through the MIT-run online platform *Lookit* (Scott and Schulz, 2017). Families were provided a website link and asked to participate in the study at a time of their choosing. Upon entering the study page, caregivers were guided on how to set up their webcam and speakers. They were given an opportunity to preview stimuli without their child present prior to beginning the task.

#### Stimuli

Visual stimuli consisted of six different animated human characters and four animated animal characters (i.e., dogs, chicken, cat, and owl). The characters were created and animated using the mobile apps *Zepeto, Talkr,* and *Animoji.* The animations were created such that the character’s mouth was moving when speech was playing.

Auditory stimuli for human trials consisted of nine different non-words (one for the task familiarization phase, and eight for the test phase). The phonemes in the non-words were selected carefully to reflect two types of words. Words with sonorous phonemes (henceforth referred to as *high talker information*) included /yom/, /yen/, /won/, and /wem/. Words with less sonorous phonemes (henceforth referred to as *low talker information*) included /gut/, /gip/, /dup/, /dit/. The selection of these words follows Andics et al. (2007), who found higher performance in talker discrimination with words that had consonants and vowels that were relatively higher in the sonority hierarchy. The auditory stimuli for animal trials consisted of recordings of animal vocalizations (i.e., sounds made by a dog, chicken, cat, and owl).

The word for the task familiarization phase was spoken by two female speakers, and the words for the test phase were spoken by four other female speakers. All speakers learned English from birth. Stimuli were recorded using the speakers’ mobile phones and edited through *Praat*. Audio files were edited to match average intensity (70 dB). Each trial consisted of a 13-s videoclip, created using a combination of *Keynote* and *iMovie*.

#### Procedure

The experiment was conducted on the family’s home computer. Caregivers were instructed to either hold their child such that the caregiver’s back and the infant’s face is facing the computer screen, or to sit beside, or behind their child with their eyes closed. Prior to the start of the study, parents were asked to ensure that the child’s face was visible through their webcam. The experiment used a preferential looking paradigm consisting of two phases: a task familiarization phase and a test phase (see Table 1 for order of task familiarization and test trials). In both phases, we included both animal and human trials. The animal trials were included to engage infants and sustain their attention. Though not the focus of the current study, infants’ performance in the animal test trials also gave us an opportunity to put their performance in human test trials into context. We predicted that infants would succeed at matching the animal sounds to the animal faces across both age groups.

**Table 1:** Summary of notes for trials in the task familiarization and test phases. Each trial was 14 seconds long. See Figure 1 for time course of audio.

Task familiarization phase	
Trials	Notes
A1, A2	There was one *Same Speaker* and one *Different speaker* trial. For each trial:
	In the first 2 seconds, two animal cartoons appeared silently side-by-side
	At the 2 second mark, one animal made a sound and wiggled
	At the 9 second mark, either the same or different animal makes a sound and wiggled. No ferns descended.
A3, A4	There was one *Same Speaker* and one *Different speaker* trial. For each trial:
	In the first 2 seconds, two animals appeared silently side-by-side
	At the 2 second mark, one animal made a sound and wiggled
	At the 9 second mark, ferns descended. Then, either the same or different animal made a sound and wiggled.
A5, A6	There was one *Same Speaker* and one *Different speaker* trial. For each trial:
	In the first 2 seconds, two human cartoons appeared silently side-by-side
	At the 2 second mark, one human made a sound and wiggles
	At the 9 second mark, ferns descended. Then, either the same or different human made a sound and wiggled.
**Test Phase**	
**Trials**	**Notes**
B1-B4	Each set of four trials had:
B6-B9	Two *Same Speaker* and one *Different speaker* trial Two *High* and two *Low talker information* trials
B11-B14	
	For each trial (see Figure 1): In the first 2 seconds, two human cartoons appeared silently side-by-side At the 2 second mark, one human made a sound. At the 9 second mark, ferns descended. Then, either the same or different human made a sound.
B16-	
B19	
B5	Across the four animal trials, there were:
B15	Two *Same speaker* and two *Different speaker* trialsFor each trial: In the first 2 seconds, two animal cartoons appeared silently side-by-side At the 2 second mark, one animal made a sound. At the 9 second mark, ferns descended. Then, either the same or different animal made a sound.
B20	
B10	

The task familiarization phase consisted of four animal and two human trials, for a total of six trials. The task familiarization phase introduced the eventual task in a gradual manner. The first two trials (A1 and A2) of the task familiarization phase began with two cartoon animals—one on each side of the screen. After a silent period (2 s), one of the animals would make a sound three times for 7 s, then wiggle slightly to indicate that they were the animal making the noise. Then, either the same or the other animal would make a sound once and wiggle (5 s). In the following two trials (A3 and A4), participants once again saw two cartoon animals. One of the animals would once again make a sound three times and wiggle. This time, a set of ferns descended to cover the animals’ mouths. After the ferns descended, either the same or the other animal would make a sound once while wiggling. For the final two task familiarization trials (A5 and A6), two females, human animations appeared—one on each side of the screen. These trials began with one of the speakers producing a one-syllable nonsense word three times while wiggling. Then, similar to the previous trials, a set of ferns descended to cover the mouths of both speakers. Finally, either the same or the other speaker would produce a one-syllable nonsense word once while wiggling. If they could learn the pairing, we expected that infants would look toward the speaker producing the word based on the vocal properties that they heard.

**Figure 1:**
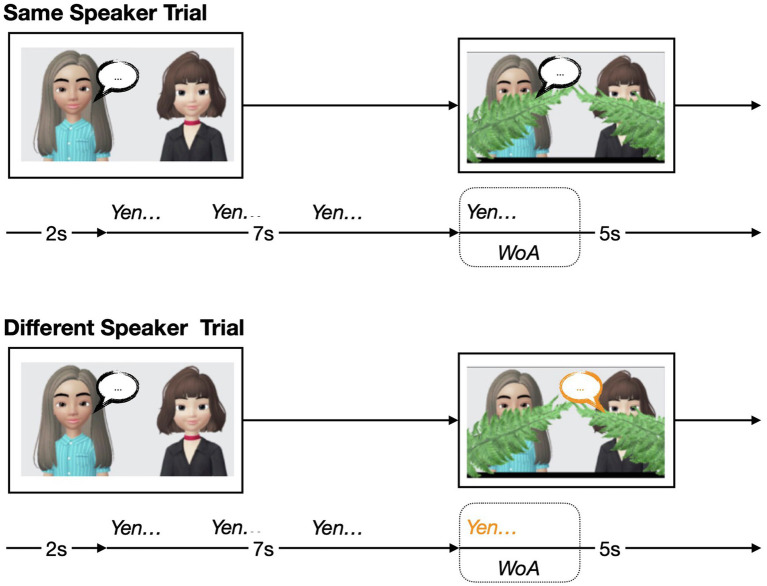
Visual schematic of the two types of trials in the test phase of the experiment: *Same Speaker* trial, and *Different Speaker* trial. ***WoA*** represents window of analysis (WoA), which was 2 s long.

The test phase consisted of four sets of five trials (four human trials, followed by one animal trial), for a total of 20 trials. In the human trials (B1–4), participants were once again presented with two female, animated humans—one on each side of the screen—and one of the speakers would produce a one-syllable nonsense word three times for 7 s. In contrast to the task familiarization trials, the speakers did not wiggle during the test trials. Then, ferns would once again descend to cover the speakers’ mouths. Once their mouths were covered, and during the critical window of analysis (WoA; 2 s), one of the speakers would produce the same one-syllable nonsense word once. The animal trials (B5) proceeded in the same way as the human trials, except that there were two cartoon animals instead of two human animations. See Figure 1 for a visual time course of the test trials.

Critically, test trials varied in two ways: trial type (*Same* vs. *Different Speaker*) and talker information (*High* vs. *Low Talker Information*). During *Same Speaker* trials, the speaker during the critical WoA was the same speaker as the one who spoke first during the trial. During *Different Speaker* trials, the other speaker would produce the same one-syllable nonsense word once. For some of the trials, the one-syllable nonsense word consisted of sonorous phonemes (*high talker information* trials). On other trials, the word consisted of less sonorous phonemes (*low talker information* trials). Each set of trials was counter-balanced, such that each set consisted of two Same Speaker and Different Speaker trials each, as well as two high talker information and low talker information trials each.

Several measures were put into place to minimize any bias that infants may have toward a particular character or stimuli. For both phases, the position of the speaker (i.e., left vs. right) was counterbalanced through the experiment. It was equally likely for each character to be the initial speaker. We also used different auditory tokens across a single trial for human trials.

Participants’ behavior was recorded *via* webcam for the duration of all trials. Participants were given two opportunities to take breaks, during which their webcam was not recording. The first break occurred after the task familiarization phase, and the second break occurred halfway through the testing phase. The breaks were not timed and the participants chose when to resume the study.

#### Data Preparation and Predictions

Based on previous work (Orena and Werker, 2021), we preset the critical WoA to be 367 ms after the critical utterance (9,367–11,367 ms). Note that the WoA was offset by 367 ms as this is the reported duration of time needed for infants to initiate an eye movement after hearing speech sounds (Swingley, 2012).

For all trials, the dependent variable was proportion looking time to the target voice of the critical utterance. Thus, a proportion looking time above 0.5 indicates that the child was proportionally looking to the correct speaker. In contrast, a proportion looking time below 0.5 indicates that the child was proportionally looking to the other speaker. A proportion looking time of 0.5 indicates that the child was looking at both speakers at chance levels. Trials in which infants looked at the screen for less than 1 s were excluded from analysis. We expected that infants would continue to look at the correct speaker during the *Same Speaker* trials. If infants were able to learn aspects of the speakers’ voices during the trial, then they should switch to also look at the correct speaker during the *Different Speaker* trials.

### Results and Discussion

#### 12-Month-Old Infants

**Figure 2:**
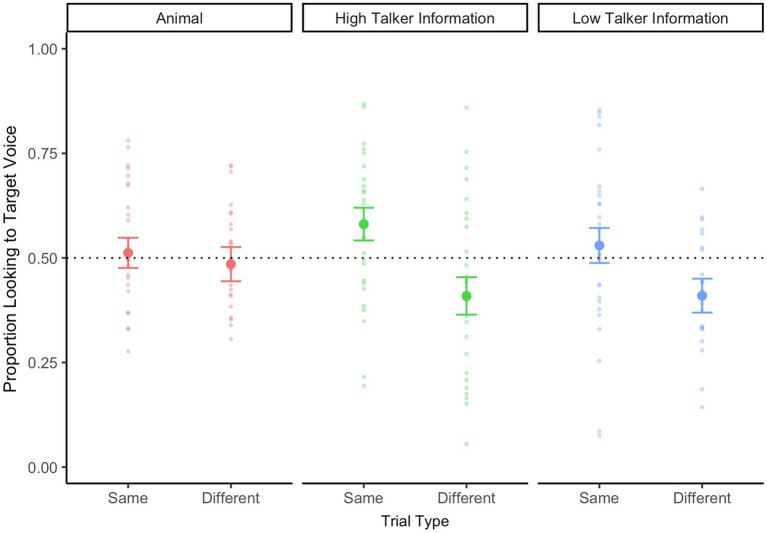
Twelve-month-old infants’ looking data during the critical WoA in the test phase, separated by trial type (*same speaker* vs. *different speaker*) and talker information (high talker vs. low talker). The looking data for animal trials are also represented on this graph. A value above 0.5 represents proportionally longer looking time to the target voice. The dotted line at 0.5 refers to equal proportion looking to both faces on the screen. Errors bars represent standard error.

Before conducting the main analysis, we first examined 12-month-old infants’ looking behaviors during the *Animal* trials. Infants’ proportion looking to target voice during the WoA is plotted in Figure 2. Surprisingly, 12-month-old infants’ were at chance levels for both *Same Speaker* and *Different Speaker* trials [*t*(24) = 0.33, *p* = 0.74, *d* = 0.07 and *t*(25) = −0.36, *p* = 0.72, *d* = 0.07, respectively].

Next, we examined whether 12-month-old infants showed any pattern of voice recognition during the main trials. We submitted infants’ proportion looking to the target voice to a repeated-measures ANOVA, with trial type (same speaker vs. different speaker) and talker information (high talker information vs. low talker information) as within-subjects’ factors. There was a significant main effect of Trial Type [*F*(1,25) = 17.27, *p* < 0.001, $\eta_{p}^{2}$ = 0.41], suggesting that infants performed differently across the two trial types, but no main effect of Talker Information [*F*(1,25) = 0.29, *p* = 0.59, $\eta_{p}^{2}$ = 0.01], nor an interaction between the two factors [*F*(1,25) = 0.36, *p* = 0.55, $\eta_{p}^{2}$ = 0.01]. *T*-tests against chance levels (0.5) indicated that during *Same Speaker* trials, infants continued to look at the correct speaker during the final part of the trial when they heard speakers produce words with *high talker information* [*t*(25) = 2.07, *p* = 0.04, *d* = 0.41], but not when they heard speakers produce words with *low talker information* [*t*(25) = 0.71, *p* = 0.48, *d* = 0.14]. During *different speaker* trials, infants did not shift to look at the correct speaker during the final part of the trial. Instead, they continued to look at the first initial speaker—whether they produced words with *high or low talker information* [*t*(25) = −2.03, *p* = 0.05, *d* = 0.40 and *t*(25) = −2.22, *p* = 0.03, *d* = 0.44, respectively].

Taken together, these looking patterns do not provide any evidence that infants were responding to the vocal stimuli during the critical part of the trial. Instead, the data suggest that infants were merely continuing to look at the initial speaker of the trial, regardless of whose voice they heard during the critical trial portion.

#### 24-Month-Old Infants

**Figure 3:**
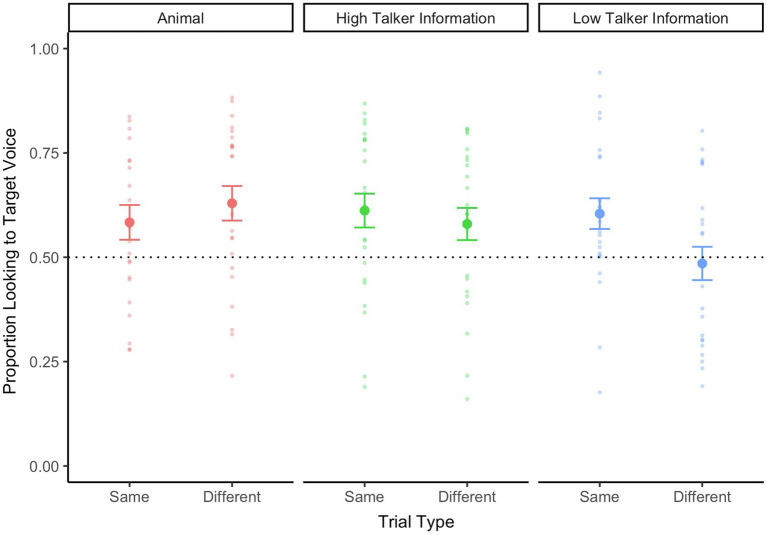
Twenty-four-month-old infants’ looking data during the critical WoA in the test phase, separated by trial type (s*ame speaker* vs. *different speaker*) and talker information (high talker vs. low talker). The looking data for animal trials are also represented on this graph. A value above 0.5 represents proportionally longer looking time to the target voice. The dotted line at 0.5 refers to equal proportion looking to both faces on the screen. Errors bars represent standard error.

Next, we examined 24-month-old infants’ looking behaviors during the experiment. Infants’ proportion looking to target voice during the WoA is plotted in Figure 3. During the *Animal* trials, 24-month-old infants’ looked toward the target animal upon hearing the animal sounds—and this was the case for both *Same Speaker* and *Different Speaker* trials [*t*(22) = 2.01, *p* = 0.05, *d* = 0.42 and *t*(22) = 3.12, *p* = 0.01, *d* = 0.65, respectively].

We then examined whether 24-month-old infants showed any pattern of voice recognition during the main trials. Similar to the earlier analysis with younger infants, we submitted 24-month-old infants’ proportion looking to the target voice to repeated-measures ANOVA, with trial type (Same Speaker vs. Different Speaker) and talker information (high talker information vs. Low Talker Information) as within-subjects factors. There were no main effects of either Trial Type [*F*(1,23) = 3.51, *p* = 0.07, $\eta_{p}^{2}$ = 0.13] or Talker Information [*F*(1,23) = 2.23, *p* < 0.15, $\eta_{p}^{2}$ = 0.09]. There was no interaction between the two factors [*F*(1,23) = 1.35, *p* = 0.26, $\eta_{p}^{2}$ = 0.06]. Nonetheless, we conducted planned comparisons against chance levels. During *Same Speaker* trials, infants continued to look at the correct speaker during the final part of the trial when they heard speakers produce words with *high talker information* [*t*(23) = 2.76, *p* = 0.01, *d* = 0.56], as well as when they heard speakers produce words with *low talker information [t*(25) = 2.84, *p* < 0.001, *d* = 0.58]. Intriguingly, during *different speaker* trials, infants shifted to look at the correct speaker during the final part of the trial when they heard speakers produce words with *high talker information* [*t*(23) = 2.06, *p* = 0.05, *d* = 0.42]. However, when they heard speakers produce words with *low talker information,* their proportion looking to both speakers was at chance levels [*t*(23) = −0.37, *p* = 0.71, *d* = 0.08].

These findings indicate that 24-month-old infants were able to learn the initial speaker’s voice when producing words with “high talker information.” They continued to correctly look at the initial speaker even after mouth movements were occluded. They also disambiguated and looked at a different speaker when they heard another voice after the ferns covered the characters’ mouths. However, they did not show the same pattern of looking behaviors when the speakers were producing words with “low talker information.”

## Discussion

In the current study, we examined the voice recognition skills of young children. Specifically, we tested infants’ ability to detect a speaker change while speakers’ faces were partially obscured. Infants’ performance in the task revealed at least two important findings.

First, our findings show that stable voice recognition skills—especially for unfamiliar voices—are present by 24 months of age. This older group of infants looked proportionally more at the target voices during the preset WoA for both *Same Speaker* and *Different Speaker* trials. These findings suggest that 24-month-old infants were able to learn some aspect of the first speaker’s voice such that: (i) they continued looking at her when they heard her voice again, even after objects blocked infants’ visual access to the speakers’ faces, and (ii) they showed a disambiguation response and looked toward another speaker when they heard another speaker’s voice.

Second, our secondary analyzes reveal that the level of unfamiliar voice learning depends, in part, on the phonetic content of the spoken words—findings that mirror those found with adults in Andics et al. (2007). The 24-month-olds showed a disambiguation response in *Different Speaker* trials only during trials when speakers were saying words with *high talker information* (i.e., tokens with sonorous phonemes). These findings confirm that, even for infants, phonetic content affects infants’ ability to learn unfamiliar voices. The results further confirm that the speech processing system that is sensitive to the integration of indexical and linguistic information is in place by late infancy (e.g., Mulak et al., 2017).

Interpreting data from the 12-month-old infants is less straightforward. Regardless of who was speaking during the critical period of the trial, 12-month-old infants continued to look at the first speaker of the trial. Interestingly, 12-month-old infants also did not show signs of recognition for animal sounds. One interpretation for these findings is that unfamiliar voice recognition is a challenging task for young infants. For example, Fecher et al. (2019) found that 16.5-month-old infants were able to learn the voices of two unfamiliar speakers, but only when the acoustic differences between the speakers were large (one male and one female speaker). When there were two speakers of the same gender, 16.5-month-old infants did not show signs of voice learning.

Why might 12-month-old infants have difficulty with the current task? Firstly, the occluding ferns may have disrupted infants’ learning of the unfamiliar voices. Indeed, visual information can help adults encode and retain aspects of a speaker’s voice (Sheffert and Olsen, 2004), and access to synchronized visual information can facilitate infants’ performance in other speech processing tasks (Hollich et al., 2005). A real-world, similar context is the use of face masks, which cover the lower half of a speaker’s face. Some have raised concerns about whether this visual occluder may compromise speech processing (Green et al., 2021). As the current study did not have an extra condition without any visual occluders, further research is needed to address this question.

Alternatively, it is possible that learning to recognize an unfamiliar voice is a challenging task for all 12-month-olds (as in Fecher et al., 2019). Indeed, voice recognition is demonstrably more difficult than voice discrimination (see Perrachione et al., 2011 for a review of talker processing tasks). The discrimination of two auditory tokens can rely on low-level mechanisms, but associating a novel face with a novel voice is more cognitively demanding. Moreover, in the current study, infants heard speakers produce only three instances of one token before being tested on their recognition of that voice. Infants may thus have needed more exposure to the initial speaker, or a less artificial experiment, to show recognition. Indeed, Fecher et al. (2019) found that learning voices is facilitated when the naturalness and social relevance of the task is increased. Nonetheless, the current study suggests that unfamiliar voice recognition is more challenging at 12 months than at 24 months.

It is important to note that the current study was conducted *via* the online platform, *Lookit* (Scott and Schulz, 2017). This platform is the first large-scale crowdsourcing platform for conducting online developmental studies. There are multitude benefits of an online platform, including the ability to efficiently run participants, to recruit more diverse participants, and to continue research during laboratory shut-downs, such as during the COVID-19 pandemic. There has been some success in validating the use of these platforms, including findings that experimental conclusions derived *via* this platform are comparable to those of in-lab studies (Scott et al., 2017; Smith-Flores et al. 2022). Note, however, that effect sizes appear to be smaller for online studies, which may also explain some of our smaller effects. Others have raised concerns about the replicability of online *Lookit* experiments (Lapidow et al., 2021), potentially due to parental interference. These concerns further temper our interpretation of the 12-month-old data, especially given that they were not successful in identifying the animals from their sounds.

To conclude, this study examined infants’ ability to learn unfamiliar voices. We found that 24-month-old infants were able to learn an unfamiliar voice sufficiently well to detect a voice change when objects blocked visual access to the speaker’s mouths. These findings are highly relevant to the current pandemic and the increased use of face masks. Particularly, these findings reveal that 24-month-old infants can encode indexical properties of an unfamiliar speaker’s voice, and they can access this information even when visual access to the speaker’s mouth is blocked. Certainly, there are important follow-ups to provide firmer conclusions. For example, face masks affect the acoustics of speech production (Llamas et al., 2008), and it would be interesting to investigate whether children’s perception of speech and voices are affected by these alterations. Moreover, one could ask whether exposure to more speakers—including more speakers with masks—might promote learning in this domain. Indeed, prior work has shown that speaker variability promotes learning in the linguistic domain (e.g., Rost and McMurray, 2009; but also see Bergmann and Cristia, 2018). Nonetheless, the successful evidence showed that infants at 24 months could recognize and learn speaker voices even when the face is partially obscured, complements the growing research showing that adults are able to adapt to mask wearing, with equal recognition of both speech and emotional expressions (Trainin and Yeshurun, 2021). Research on these and related topics will help to improve our understanding of how infants make use of the multisensory information around them to adapt to different contexts.

## Data Availability Statement

The raw data supporting the conclusions of this article will be made available by the authors, without undue reservation.

## Ethics Statement

The studies involving human participants were reviewed and approved by the University of British Columbia, Behavioural Research Ethics Board. Written informed consent to participate in this study was provided by the participants’ caregiver.

## Author Contributions

AO, AM, and JW contributed to the conception and design of the study. AM created the stimuli set, coded the looking data, with support from other research assistants, and wrote a section of the manuscript. AO and AM set up the study on *Lookit*. AO performed the statistical analysis, with guidance from JW, and wrote the first draft of the manuscript. All authors contributed to the article and approved the submitted version.

## Funding

This research was funded by the Social Sciences and Humanities Research Council of Canada (grants 435-2019-0306 and 895-2020-1004) to JW, an NSERC Undergraduate Student Research Award to AM, and a Fonds de Recherche du Québec—Nature et Technologies Postdoctoral Fellowship to AO.

## Conflict of Interest

The authors declare that the research was conducted in the absence of any commercial or financial relationships that could be construed as a potential conflict of interest.

## Publisher’s Note

All claims expressed in this article are solely those of the authors and do not necessarily represent those of their affiliated organizations, or those of the publisher, the editors and the reviewers. Any product that may be evaluated in this article, or claim that may be made by its manufacturer, is not guaranteed or endorsed by the publisher.
